# Independent, Rapid and Targeted Loss of Highly Repetitive DNA in Natural and Synthetic Allopolyploids of *Nicotiana tabacum*

**DOI:** 10.1371/journal.pone.0036963

**Published:** 2012-05-14

**Authors:** Simon Renny-Byfield, Ales Kovařík, Michael Chester, Richard A. Nichols, Jiri Macas, Petr Novák, Andrew R. Leitch

**Affiliations:** 1 School of Biological and Chemical Sciences, Queen Mary University of London, London, United Kingdom; 2 Institute of Biophysics, Academy of Sciences of the Czech Republic, Brno, Czech Republic; 3 Laboratory of Molecular Systematics and Evolutionary Genetics, Florida Museum of Natural History, University of Florida, Gainesville, Florida, United States of America; 4 Biology Centre ASCR, Institute of Plant Molecular Biology, České Budějovice, Czech Republic; University of Warwick, United kingdom

## Abstract

Allopolyploidy (interspecific hybridisation and polyploidy) has played a significant role in the evolutionary history of angiosperms and can result in genomic, epigenetic and transcriptomic perturbations. We examine the immediate effects of allopolyploidy on repetitive DNA by comparing the genomes of synthetic and natural *Nicotiana tabacum* with diploid progenitors *N. tomentosiformis* (paternal progenitor) and *N. sylvestris* (maternal progenitor). Using next generation sequencing, a recently developed graph-based repeat identification pipeline, Southern blot and fluorescence *in situ* hybridisation (FISH) we characterise two highly repetitive DNA sequences (*Nic*CL3 and *Nic*CL7/30). Analysis of two independent high-throughput DNA sequencing datasets indicates *Nic*CL3 forms 1.6–1.9% of the genome in *N. tomentosiformis*, sequences that occur in multiple, discontinuous tandem arrays scattered over several chromosomes. Abundance estimates, based on sequencing depth, indicate *Nic*CL3 is almost absent in *N. sylvestris* and has been dramatically reduced in copy number in the allopolyploid *N. tabacum*. Surprisingly elimination of *Nic*CL3 is repeated in some synthetic lines of *N. tabacum* in their forth generation. The retroelement *Nic*CL7/30, which occurs interspersed with *Nic*CL3, is also under-represented but to a much lesser degree, revealing targeted elimination of the latter. Analysis of paired-end sequencing data indicates the tandem component of *Nic*CL3 has been preferentially removed in natural *N. tabacum*, increasing the proportion of the dispersed component. This occurs across multiple blocks of discontinuous repeats and based on the distribution of nucleotide similarity among *Nic*CL3 units, was concurrent with rounds of sequence homogenisation.

## Introduction

Polyploidy, where an individual possesses more than a diploid complement of chromosomes, is a fundamental process in the evolution of land plants [1], [2], [3], [4]. Molecular evidence suggests a whole genome duplication (WGD) at the base of all seed plants and another at the base of the angiosperms, and with many lineages having additional WGDs events in their ancestry [5]. Many of these polyploid events are associated with major radiations of land plants [3], [5].

The phenomenon of polyploidy is often associated with interspecific hybridisation (allopolyploidy), where divergent genomes are unified within a single nucleus. It has been suggested that this process can induce rapid, reproducible and directional changes to the progenitor sub-genomes [6], [7], [8], [9], [10], [11]. Analysis of wheat F_1_ hybrids has revealed preferential loss of sequences from one of the progenitor genomes, as well as reproducible loss of DNA sequences across independently synthesised neo-tetraploids [12]. For example second-generation neo-tetraploids of a cross between *Aegilops tauschii* × *Triticum turgidum* have shown elimination of a sequence derived from *A. tauschii* in a tissue specific manner, likely to have occurred during embryo development [13]. Similarly, abundance estimates for repetitive DNA in the genomes of the allopolyploid *Nicotiana tabacum* (formed 20,000–200,000 years ago) and its diploid progenitors indicate the preferential elimination of paternally derived DNA, contributing to genome downsizing thought to have occurred in this species [14], [15]. A comparable pattern is observed in synthetic *N. tabacum* Th37 lines, produced in the 1970s [16].

The emergence of high throughput DNA sequencing [17] has allowed the analysis of highly repetitive sequences in the genomes of several angiosperm species including banana, pea, soybean, barley, *Silene latifolia* as well as allopolyploid *N. tabacum* and its diploid progenitors [15], [18], [19], [20], [21], [22]. Here we examine *Nicotiana tabacum* and progenitors *N. sylvestris* (maternal S-genome donor) and *N. tomentosiformis* (paternal T-genome donor) focusing on the genomic organisation and abundance of two novel repeat families, *Nic*CL3 and *Nic*CL7/30. We used high throughput DNA sequencing to determine if these repeats are inherited in an additive manner, and to assess any changes in their organisation following allopolyploidy.

## Materials and Methods

### Plant material

The following accession were used: [1] *Nicotiana tabacum* cv. SR1 Petit Havana and cv. 095-55. [2] *Nicotiana sylvestris* Speg. & Comes ac. ITB626 both originating from the Tobacco Institute, Imperial Tobacco Group, Bergerac, France. [3] *Nicotiana tomentosiformis* Goodsp. ac. NIC 479/84 (Institute of Plant Genetics and Crop Plant Research, Gatersleben, Germany), TW142 (USDA, North Carolina State University, Raleigh, NC, USA) and Nee et al. 51771 (New York Botanic Gardens). [4] Synthetic *N. tabacum* Th37 lines in generation four, generated by Burk [23] and characterized previously [16], [24]. [5] Synthetic *N. tabacum* TR1-A in generation S0, generated and characterized by Lim *et al.* [25]. [6] Diploid species in section *Tomentosae* [26] derived from USDA: *N. tomentosa* Ruiz and Pav.; *N. kawakamii* Y. Ohashi; *N. otophora* Griseb., *N. setchelli* Goodsp.; and *N. glutinosa* L. (section *Undulatae*, formerly section *Tomentosae* [27], [28].

### High-throughput sequencing of genomic DNA

We used Roche 454 FLX pyrosequencing (454 sequencing) as generated in Renny-Byfield *et* *al*. [15]. Sequence reads are deposited in the NCBI sequence read archive (SRA) under the study accession number SRA023759. We sequenced here, using the Illumina Genome Analyzer xII at The Genome Centre Queen Mary University of London, between 47–61% of the genomes of *N. tomentosiformis* (ac. NIC 479/84), *N. sylvestris* (ac. ITB626), *N. tabacum* (ac. SR1) and the synthetic *N. tabacum* TR1-A line (details of the sequencing output can be found in Table S1; sequence reads were submitted to the NCBI SRA under the study accession number: SRA045794). We choose the *N. tomentosiformis* accession NIC 479/84 because it most closely resembles the T-genome of *N. tabacum* [29], [30]. There is no *N. sylvestris* accession that is considered to be more closely related to the *N. tabacum* S-genome than any other [30].

### Clustering, contig assembly and sequence analysis

**Figure 1:**
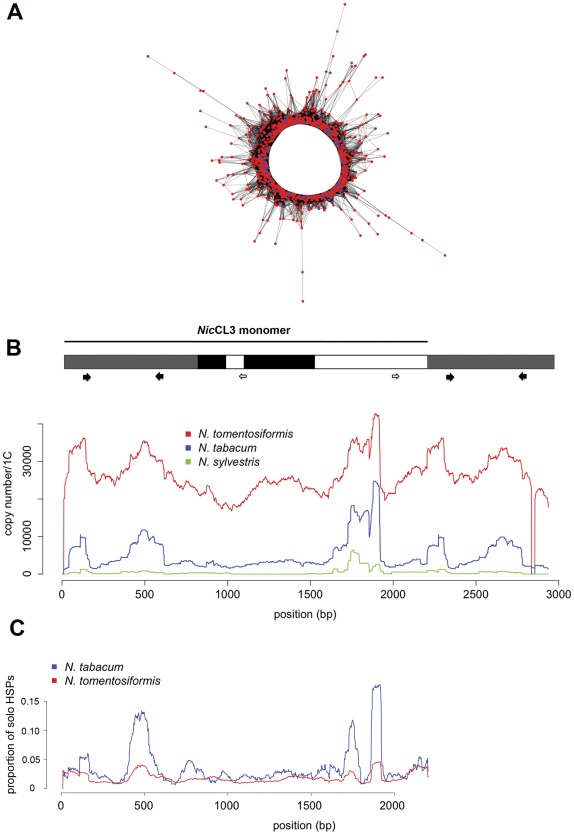
Structure and copy number of *Nic*CL3. (a) Graphical 2D projection of a three dimensional network where each node represents a single 454 sequence within *Nic*CL3. Nodes are placed according to sequence similarity, where similar sequences are placed close together, and more distantly related sequences further away. Sequence similarity is indicated by edges (connecting lines). Red nodes represent sequence reads originating from *N. tomentosiformis* and blue are reads originating from *N. tabacum*.(b) A diagrammatic representation of the consensus sequence of the most abundant contig (contig 8) of CL3, here called NicCL3. The line (top) indicates the NicCL3 monomer, the greyed regions represents those regions of the contig that are repeated because it contains part of a second monomer. Copy-number estimates (estimated by 454 read-depth) for allopolyploid N. tabacum and the progenitor diploids are shown. The approximate positions of primer sets 1 (black arrows) and primer set 2 (open arrows) are shown (see Experimental Procedures). Regions in NicCL3 matching the d and j-locus found flanking a endogenous pararetrovirus (NtoEPRV) described in [42] are highlighted in black. (c) Paired-end reads were used to determine the occurrence of dispersed NicCL3 sequence and/or insertion of other sequences within NicCL3. The proportion of solo HSPs (NicCL3 sequences whose paired read does not match NicCL3) is shown mapped along the monomer of NicCL3 contig 8 for N. tabacum and N. tomentosiformis. Note there are regions along the monomer that are more likely to be associated with sequences other than NicCL3 (solo HSPs) and that the proportion of solo HSPs is considerably higher in N. tabacum.

**Figure 2:**
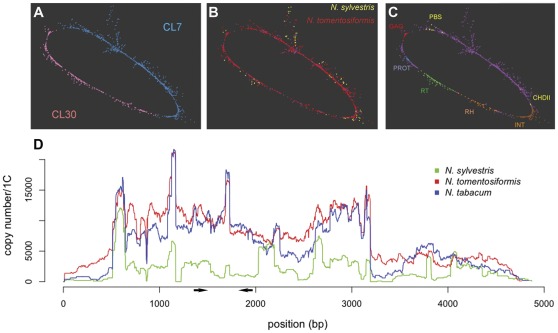
The cluster *Nic*CL7/30. (**a**) Cluster *Nic*CL7/30 shown as a graph. Individual sequence reads are represented as nodes on the graph and for simplicity edges representing similarity hits are not shown. The position of nodes was calculated using the Fruchterman-Reingold algorithm. (b) The same graph but with sequences highlighted depending on the progenitor species from which they derive. (c) Another representation of NicCL7/30 but indicating sequence similarity to conserved coding domains (CCD) including protease (PROT), reverse transcriptase (RT), RNaseH (RH), integrase (INT), chromovirus chromo-domain (CHDII) and gag-pol (GAG). (d) Estimated copy-numbers from 454 read-depth analysis, along the length of the most abundant contig in the merged cluster NicCL7/30. A region between ∼500 and 3200 bp is more abundant than the remaining contig and likely represents the LTR region of this retroelement, where higher abundance may be due to the presence of solo-LTRs. The position of PCR primers used to make probes for this sequence are indicated with arrows.

A graph-based clustering approach described in was used to identify and reconstruct, *in silico*, the major repeat types present in the genomes of *N. tabacum*, *N. sylvestris* and *N. tomentosiformis* as described in Renny-Byfield *et* *al*. [15]. A combined dataset of 454 sequence reads from all three species was used to generate clusters and contigs representing repetitive DNA sequences. Mutual similarities can then be visualised in graph form (Fig. 1 a and Fig 2 b) in which nodes correspond to sequence reads, and a Fruchterman-Reingold algorithm is used to position nodes. Reads that are most similar are placed closest together whilst those that are less closely related are more distal (described in detail in Novak *et* *al*. [31]). Contig assembly is performed with reads from each cluster and the contigs are named according to the number of the cluster from which they derive (X) and *Nic* designates *Nicotiana*, i.e. *Nic*CLX. Each cluster typically generates multiple contigs, each of which is designated a number (Y), giving a format *Nic*CLX contigY. All contigs assembled in this work are available via our websites: http://webspace.qmul.ac.uk/sbyfield/Simon_Renny-Byfield/Data.html and http://webspace.qmul.ac.uk/arleitch/Site/Home.html.

We estimated the genomic abundance of all contigs in each cluster using the “map reads to reference” function of CLC Genomics workbench version 4, requiring 80% sequence similarity over 50% of the sequencing read (any given sequence from the Illumina or Roche 454 datasets was mapped only once). The depth at which reads are mapped reflects the genomic proportion (GP) of the corresponding repeat and so provides a measure of its abundance within the genome. To obtain the GP of a given cluster, all GPs for contigs within that cluster were summed. For Roche 454 and Illumina datasets the average read-depth along each contig (RD), genome representation (GR, calculated as RD × contig length) and genome proportion (GP, calculated as (GR/total size of the dataset in base pairs) × 100) were calculated for each species independently. For the synthetic *N. tabacum* line TR1-A only Illumina sequence reads were used to calculate GP.

Clusters were then subjected to sequence similarity searches against RepBase [32] in order to identify, where possible, the repeat type from which they derive.

### Analysis of *Nic*CL3 using paired-end reads

We analysed paired-end Illumina data from *N. tomentosiformis* and *N. tabacum* to assess the occurrence of sequences where one of the paired reads hits *Nic*CL3 and the partner read did not. Reads were screened for quality and both reads of the pair were removed from the dataset if one or other of the reads failed the following quality checks: the read was at least 95 bp long and with no more than five unidentified nucleotides (Ns). All reads passing the quality checks were then trimmed to 95 bp in length. Illumina reads were subjected to similarity searches (requiring 90% sequence similarity along 55% of the sequence read) against contig 8 of *Nic*CL3. More stringent settings were used in this instance to compensate for the shorter read length of Illumina reads. The proportion of pairs where both reads hit (termed a dual High-scoring Segment Pair (HSP)) was recorded. Subsequently unmatched sequences from a pair, where only one read matches contig 8 of *Nic*CL3 (termed solo HSPs), were subjected to further sequence similarity searches to all other clusters. Those that hit other contigs in the *Nic*CL3 cluster were reassigned as dual HSPs. The distribution of solo HSPs was then plotted as a proportion of total HSPs along the length of the monomer of contig 8 of *Nic*CL3.

### Sequence similarity in *Nic*CL3

We compared sequence similarity of *Nic*CL3 derived sequences in *N. tomentosiformis* and *N. tabacum* using 454 reads described in Renny-Byfield *et* *al*. [15]. Reads deriving from *N. tomentosiformis* and *N. tabacum* were analysed by BLASTn analysis using the stand alone BLAST program [33] with default parameters with the exception of the following: -e 1e^−5^, -v 80,000, -b 80,000, -F F. Reads from each species were analysed separately in a pair-wise fashion. Custom BioPerl scripts were used to extract the sequence similarity of all hits to a given read (excluding the query sequence hitting itself). In addition we analysed a mix of all the *Nic*CL3 derived reads from both of the progenitor species. Pair-wise similarity scores for *Nic*CL3 sequences from *N. tabacum* and *N. tomentosiformis* and the mix of the progenitor species were plotted as frequency distributions and density estimates using the R statistical package [34].

We used BLASTn to analyse the proportion of *Nic*CL3 reads that matched the consensus sequence (*Nic*CL3, contig 8) at any given nucleotide for *N. tomentosiformis* and *N. tabacum* using custom BioPerl scripts.

### PCR

DNA was amplified from 50 ng of *N. tomentosiformis* (ac. NIC 479/84) genomic DNA using Bioline *Taq* DNA polymerase (San Francisco, USA) supplemented with 1× Bioline NH_4_ Buffer, 1.5 mM MgCl_2_, 0.2 mM of each dNTP and 0.2 µM of each primer pair.

- Primer pair 1 (forward: 5′-GGTAGAGTAGTGATGAGG-3′ reverse: 5′-TGGTGGATTAAGGATTGG-3′, Fig. 1 b, filled arrows). PCR primers were designed from *Nic*CL3 contig 8. PCR involved an initial denaturation step of 3 min at 94°C, followed by 36 cycles of 94°C for 40 s, 48°C for 40 s and 72°C for 45 s, followed by a final extension step of 72°C for 3 min.
- PCR analysis with primer pair 2 (5′-TAAAACTCCCAACATCCG-3′ and reverse 5′-TGGGTATAGTGAAGACGA-3′, Fig. 1 b, open arrows). PCR primers were designed against a second region of *Nic*CL3 contig 8. PCR used an initial denaturation of 3 min at 94°C, followed by 36 cycles of 94°C for 50 s, 48°C for 1 min and 72°C for 3 min, followed by a final extension of 72°C for 7 min.
- Primer pair 3 (forward 5′-TGTGTTGGGCTGTTTTGT' and reverse 5′-CTTGCTGCTCTCTTGACT-3′). PCR primers were designed against *Nic*CL7 contig 7. PCR followed that described for primer pair 1.

### Cloning and sequencing

PCR products of *Nic*CL3 and *Nic*CL7 were cleaned using the Qiagen PCR purification kit and cloned using a TOPO® cloning kit with the pCR®2.1 vector by Invitrogen according to the manufacturers instructions. Positive clones were sequenced using T7 forward and M13 reverse primers at Eurofins MWG|operon. The clones sharing highest similarity with the appropriate contig were selected and used to produce probes for fluorescent *in situ* hybridisation (FISH) and Southern blot hybridisation.

### Probes for FISH

(1) Probes were prepared from a clone (number 9; NCBI accession JQ899200) of *Nic*CL3 (using primer pair 1) and from a clone (number 1; NCBI accession JQ899201) of *Nic*CL7 (using primer pair 3). PCR amplification used the conditions described above with the addition of 0.5 mM digoxigenin-11-dUTP or biotin-16-dUTP labelled nucleotides. All probes were cleaned using Qiagen PCR Purification Kit according to the manufacturers instructions.
(2) An 18S nuclear ribosomal DNA (rDNA) sequence cloned from *Allium cernum* [35] was used to generate a probe as detailed above, with the exception that the extension step of the PCR was at 72°C for 2 min and the final extension was for 7 min. The following primers were used, 18S2F 5′-CGGAGAATTAGGGTTCGATTC-3′ and AB101R 5′-ACGAATTCATGGTCCGGTGAAGTGTTCG-3′, the latter modified from Sun *et* *al.* [36].
(3) Total genomic DNA for genomic *in situ* hybridisation (GISH) from *N. tomentosiformis* (ac. NIC 479/84) and *N. sylvestris* was labelled with biotin-16-dUTP and digoxigenin-11-dUTP, respectively, by using the Roche Nick Translation Kit according to the manufacturers instructions.

### Fluorescence *in situ* hybridisation (FISH)

Metaphases were accumulated in freshly harvested root-tips by pre-treatment in saturated Gammexane® (hexachlorocyclohexane, Sigma) in water for 4 h. Root-tips were fixed for 24 h in 3∶1 absolute ethanol:glacial acetic acid and stored at −20°C in 100% ethanol. Root-tip metaphases were spread onto glass slides after enzyme digestion as described in Lim *et* *al.* [37], and checked for quality using phase contrast microscopy.

FISH followed the protocol described in Lim *et* *al*. [38]. Briefly, probe DNA (delivering 50 ng of cloned probe or 100 ng of genomic probe per slide) was added to the probe hybridisation mix (50% (v/v) formamide, 10% (w/v) dextran sulphate, 0.1% (w/v) sodium dodecyl sulphate in 2x SSC (0.3 M NaCl, 0.03 M sodium citrate, pH 7.0)). About 50 µl of the probe mixture was added to each chromosomal preparation and the material denatured with a dyad slide heating-block at 70°C for 2 min. After overnight hybridisation at 37°C, slides were washed in 20–25% (v/v) formamide in 0.1x SSC at 42°C at an estimated hybridisation stringency of 85–89%. Sites of probe hybridisation were detected with 20 µg.ml^−1^ fluorescein conjugated anti-digoxigenin IgG (Roche Biochemicals Ltd.) and 5 µg.ml^−1^ Cy3 conjugated streptavidin (Amersham Biosciences). Chromosomes were counterstained using Vectashield with DAPI (4′,6-diamidino-2-phenylindole; Vector Laboratories). Material was photographed using a Hamamatsu Orca ER camera and a Leica DMRA2 epifluorescent microscope. Images were processed with Improvision Openlab software and Adobe Photoshop CS2, adjusting for colour balance, contrast, and brightness uniformly.

For multiple probe labelling, preparations were striped of probe and signal by a 10 min wash at 110% stringency (60% v/v formamide, 0.1x SSC at 42°C). Slides were checked to ensure no signal could be visualised. Slides were then subjected to a second round of FISH using alternative probes and re-photographed.

### Southern blot hybridisation

DNA was extracted from fresh young leaves according to Kovarik *et* *al*. [39], digested with restriction endonucleases (5 U μg^−1^ DNA, twice for 6 h), fractionated by gel electrophoresis and transferred to GE-Healthcare Hybond XL membranes using alkaline capillary transfer. Membranes were hybridized with ^32^P-labelled DNA probe (DecaLabel DNA Labeling Kit, MBI Fermentas). Southern blot hybridisation was carried out in a 0.25 M sodium phosphate buffer (pH 7.0) supplemented with 7% (w/v) sodium dodecyl sulphate (SDS) at 65°C [40]. Membranes were washed with 2x SSC, 0.1% SDS (twice for 5 min) and then with 0.2x SSC and 0.1% SDS (twice for 15 min at 65°C). The membranes were exposed to a Storage Phosphor Screen, scanned (Storm, GE-Healthcare) and the signal was quantified using Image Quant (GE-Healthcare). The DNA probe was a ∼500 bp insert of clone 9 of *Nic*CL3 used in the FISH experiments.

All materials and data are available on request.

## Results

### Clustering, contig assembly and repeat abundance estimates

A combined dataset of 454 reads from the three *Nicotiana* species totalling >70 Mb of DNA was subjected to a clustering based repeat identification procedure as described in the Materials and Methods section, and in detail in Novak *et al*. [31]. Briefly sequence reads are subjected to pair-wise sequence similarity analysis where related sequences are grouped into clusters. These clusters correspond to families of repetitive DNA sequences and the reads therein are further assembled into contigs. The depth at which Roche 454 or Illumina reads map to these sequences allows estimation of genomic proportion (GP) of the corresponding repeat. Moreover *N. tabacum* is a symmetrical hybrid since both ancestors have roughly the same genome size (∼2,650 Mbp/1C [41]). Therefore for a uniparentally inherited repeat, the expected genome proportion (GP) in *N. tabacum* is 0.5 of the parental GP.

Read-depth analysis revealed two clusters (*Nic*CL3 and *Nic*CL7) to be highly abundant in the genome of *N. tomentosiformis*. Illumina sequencing read-depth across *Nic*CL3 indicates a genome proportion (GP) of 1.60%, while similar analysis with 454 data indicate a GP of 1.91%. The corresponding values in *N. tabacum* are 0.10 and 0.09% respectively, both markedly lower than the abundance (0.80%/0.95%) that would be predicted given additivity of the parents (Table 1).

**Table 1:** Estimated abundance of two families of repetitive DNA sequences in the genomes of *N. tabacum* and progenitor species *N. sylvestris* and *N. tomentosiformis*.

		Abundance of cluster: % of genome (454/Illumina estimation)				
cluster name	most abundant contig (length in bp)	*N. tomentosiformis*	*N. sylvestris*	parental additivity	*N. tabacum*	TR1-A(S0 synthetic tobacco)
*Nic*CL3	contig 8(2926)	1.91/1.60	<0.01/<0.01	0.95/0.80	0.10/0.09	NA/0.77
*Nic*CL7/30	CL7/contig 7(4759)	1.40/1.27	0.15/0.20	0.78/0.74	0.52/0.56	NA/0.71

A graphical representation of sequence relationships in the cluster containing *Nic*CL3 is shown in Figure 1 A. Reads form a circle-like pattern indicative of direct terminal or tandem repeats. *Nic*CL3 is a tandem repeat (see below). With this graphical analysis, tandem repeats often have a globular shape in 3D-networks, particularly if the monomer size is small. The reason that *Nic*CL3 does not have this pattern is due to its length (2.2 kb). Not all of the ∼360 bp reads that make up the graph share sequence similarity (i.e. reads in different regions of the monomer will not overlap, as with a short monomer). The read connections (edges) are largely ‘linear’ until reaching either end of the monomer, where reads can bridge adjacent monomers, forcing the ends of the network to close up in a wheel like pattern. Copy number estimates along the most abundant contig (8) in the cluster *Nic*CL3 are shown in Figure 1 B. A MGBLAST search was conducted using the consensus NicCL3 monomer as a query to N. tabacum genome survey sequences (GSSs) (e-value < 1e-15). This produced 741 hits along the whole length of the NicCL3 monomer, with 381 hits showing 95% to 100% similarity, supporting the restriction digest, sequencing and clustering/assembly data.

Sequence similarity searchers of *Nic*CL3 to RepBase returned a small region (positions 717–953 with 40% amino acid identity) with similarity to GYPSODE1_I a Ty3/*gypsy*-like retroelement identified in *Solanum demissum* [32] while searches against the Pfam conserved protein domain database returned no matches. Regions with similarity to *Nicotiana tomentosiformis* endogenous pararetrovirus (NtoEPRV) insertion sites [42] were identified and indicated in black (Fig. 1 b).

The *Nic*CL7 cluster is closely related to cluster 30 (*Nic*CL30) and they are likely derived from the same repeat family. Therefore, they were merged in to a single cluster, hereafter called *Nic*CL7/30, shown graphically to be circle-like (Fig. 2 a–c). Protein BLAST searches indicate that reads within *Nic*CL7/30 have sequence similarity to reverse transcriptase (RT), integrase (INT), RNaseH (RH), protease (PROT) and GAG domains of LTR retroelements, as well as a chromovirus specific chromatin-remodeling domain (CHDII). We therefore suggest *Nic*CL3/CL30 is likely to be a chromoviruses-like (Ty3/gypsy retroelements, 70% amino acid identity along 297 bp) family of repetitive DNA, although the repetitive sequence is not formally classified. Reference sequences for *Nic*CL3 and *Nic*CL7/30 are available at the following websites: http://webspace.qmul.ac.uk/sbyfield/Simon_Renny-Byfield/data.html and http://webspace.qmul.ac.uk/arleitch/Site/Home.html.

We analysed Illumina paired-end data to assess the proportion of paired sequences where one read hits *Nic*CL3 and the other member of the pair did not (solo HSPs). In *N. tomentosiformis* and *N. tabacum*, 3.16% and 8.80% of paired reads had only one match (solo HSPs) to the *Nic*CL3 respectively. In *N. tomentosiformis* we observed 95 instances where one sequence of a pair matched *Nic*CL3 while the other matched *Nic*CL7/30. In *N. tabacum* comparisons of the distribution of solo HSPs along the length of *Nic*CL3 revealed regions of the sequence with high proportions of solo HSPs (Fig. 1 c), a similar pattern was observed in *N. tomentosiformis*, although it was less apparent. It is noteworthy that the irregular profile of copy number estimates along *Nic*CL3 corresponds closely with the distribution of solo HSPs (compare Fig. 1 b with 1 c).

### Cloning regions of *Nic*CL3 and *Nic*CL7

PCR using primer pair 1 (thick black arrows in Fig. 1 b) against the consensus of *Nic*CL3 amplified the region between position 109 and 581 bp. Cloning of the PCR product resulted in four sequences sharing between 92–96% identity with the *in silico* consensus. PCR using primer pair 3 against the region between 1488 and 1926 bp of *Nic*CL7 produced a band of the expected size. The PCR products were cloned and five clones chosen for sequencing, each had sequence similarity varying between 92 and 96% against the *in silico* consensus. Clone 9 for *Nic*CL3 and clone 1 for *Nic*CL7 were chosen for further analysis.

### FISH

**Figure 3:**
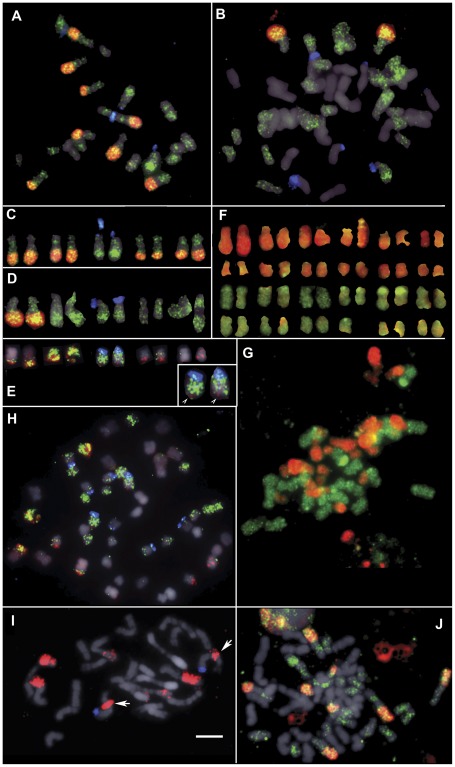
FISH of *Nic*CL3 and *Nic*CL7/30. Fluorescence *in situ* hybridisation (FISH) to metaphase chromosomes of (a, c) *N. tomentosiformis* (ac. NIC 479/84); (b, d) Th37-3; (e, h) *N. tabacum* (ac. 095-55); (i) *N. kawakamii* and; (j) TR1-A. The probes used were 18S rDNA (blue; a-e and h-i only), NtCL7 (green) and *Nic*CL3 probes (red) counter stained with DAPI (grey). Inset (e) shows enlarged chromosome T3 with *Nic*CL3 signal at the distal end of the long arm (arrow heads). (f, g) Genomic *in situ* hybridisation (GISH) to chromosomes of Th37-3, showing the *N. tomentosiformis* sub-genome (red) and *N. sylvestris* sub-genome (green). (i) Note that chromosome 3 of *N. kawakamii* (18S rDNA bearing) has a large *Nic*CL3 signal proximal to the centromere (arrows). (j) TR1-A an S0 synthetic *N. tabacum* with the expected number of *Nic*CL3 (red) signals and highly localised NtCL7 (green) signals. Scale bar is 5 µm.

FISH using the *Nic*CL3 clone 9 to metaphase spreads of *N. tomentosiformis* (ac. NIC 479/84 and Nee et al. 51771) reveals loci on eight of the large sub-metacentric chromosomes (Fig. 3 a, c and Table 2). The signal is highly localized and is exclusive to the distal region of the long arm of four chromosome pairs. The 18S rDNA-bearing chromosome (chromosome 3, following the nomenclature of Lim *et* *al*. [43]) lacks any detectable signal. In contrast there is *Nic*CL3 signal at an interstitial locus on the orthologous 18S rDNA-bearing chromosome of the diploid relative *N. kawakamii* (Fig. 3 i). *Nic*CL3 signal is also observed on chromosome T3 of *N. tabacum*, although it is restricted to the most distal regions of the long arm (boxed in Fig. 3 e). All *Nic*CL3 loci in *N. tabacum* are noticeably smaller than those in the progenitor *N. tomentosiformis* and the diploid *N. kawakamii*.

**Table 2:** Repetitive DNA family *Nic*CL3 in the genomes of *Nicotiana tabacum*, *N. tomentosiformis* and members of the section *Tomentosae*.

accession	Southern hybridisation	number of FISH signals
*N. tomentosiformis* (TW142)	+	n.s
*N. tomentosiformis* (NIC 479/83)	+ (Fig. 3)	8 (Fig. 3 a, c)
*N. tomentosiformis* (Nee et al. 51771)	n.s	8
*N. tabacum* (SR1)	+ (Fig. 3)	8
*N. tabacum* (095-55)	+	8 (Fig. 3 h, e)
Th37^a^ 1	–	n.s
3	+	2 (Fig. 3 b, d)
5	+ (Fig. 3)	n.s
6	+	n.s
7	+	2
8	+	n.s
9	n.s	–
14	n.s	2
TR1-A	n.s	8 (Fig. 3 j)
*N. sylvestris*	–	–
*N. kawakamii*	+	8 (Fig. 3 i)
*N. otophora*	–	–
*N. tomentosa*	trace amounts	–
*N. setchellii*	trace amounts	n.s
*N. glutinosa*	–	n.s

+Indicates a ladder like pattern following restriction digestion and Southern blot analysis.

n.s not screened.

–no signal detected.

a Groups of Th37 plants as described in [24].

Metaphase chromosomes of several synthetic *N. tabacum* lines (Th37-3, -7 and -14) reveal only two *Nic*CL3 signals, on a single pair of large submetacentric chromosomes (Fig. 3 b, d, Table 2). The loss of signal is not caused by the absence of *N. tomentosiformis*-derived chromosomes as GISH to metaphase spreads of Th37-3 reveal a full complement of *N. tomentosiformis* chromosomes (24 red chromosomes in Fig. 3 f, g). The S0 generation synthetic *N. tabacum* TR1-A has eight *Nic*CL3 signals as expected (Fig. 3 j).

*Nic*CL7 has a dispersed signal on all *N. tomentosiformis* chromosomes (Fig. 3 a), although some regions bind the probe more efficiently producing a band-like pattern on large submatacentric chromosome pairs, particularly evident on the 18S rDNA-bearing chromosome. *Nic*CL7 signal is associated with all *Nic*CL3 signals in *N. tomentosiformis*, Th37 and TR1-A. Th37-3 has *Nic*CL7 signal on 24 of the 48 chromosomes (Fig. 3 j); it is likely these derive from *N. tomentosiformis*. We were unable to detect any signals of *Nic*CL3 and *Nic*CL7 in *N. sylvestris* (data not shown).

### Southern blot hybridisation

**Figure 4:**
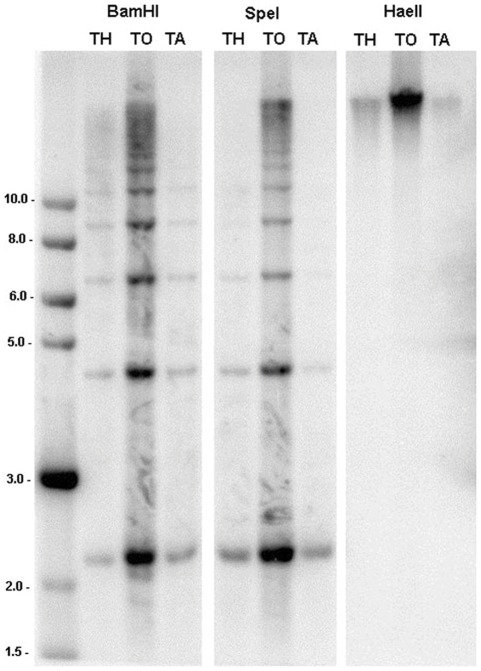
Tandem arrangement of *Nic*CL3. Southern blot hybridisation of genomic DNA from (TH) synthetic tobacco Th37-5, (TO) *N. tomentosiformis ac*. NIC 479/84 and (TA) *N. tabacum ac*. SR1 digested with *Spe*I, *Bam*HI and *Hae*II (a methylation sensitive isoschizomer of *Bam*HI) and probed with *Nic*CL3. Size indicators on the left are in kb. Digestion with *Bam*HI and *Spe*I results in a ladder like pattern, typical of tandem repeats. Digestion is inhibited when using *Hae*II, indicating extensive CG methylation of tandem units.

Southern blot hybridisation was carried out using *Nic*CL3 as a probe. For each species 1–2 µg of genomic DNA was digested with *Bam*HI and *Spe*I enzymes (Fig. 4, Table 2), which have a single restriction site within *Nic*CL3. A ladder pattern of bands was evident in *N. kawakamii*, *N. tomentosiformis* (TW142 and NIC 479/84), natural *N. tabacum* (095-55 and SR1), synthetic *N. tabacum*, Th37-3, 5, 6, 7 and 8. The bands are indicative of tandemly arranged satellite repeats arranged head to tail. The fastest migrating band corresponded to the satellite monomer (2.2 kb), contained within the 2.9 kb *in silico* reconstruction (Fig. 1 b). There was no signal detected in Th37-1, *N. sylvestris N. glutinosa* or *N. otophora*. Other species (*N. setchellii* and *N. tomentosa*) have trace amounts of background signal but lack any detectable ladder pattern (Table 2).

In natural *N. tabacum*, Th37 and *N. tomentosiformis* digestion of the unit is inhibited when the methylation sensitive restriction enzyme *Hae*II is used (with one restriction site in the monomer), indicating cytosine methylation of *Nic*CL3 at the restriction site in these species (Fig. 4).

The *in silico* consensus of *Nic*CL3 sequence includes terminal repeats (Fig. 1 B) and to confirm that these arise because the consensus includes a whole monomer and part of a second monomer in the tandem array, we designed PCR primer pair 2 (open arrows in Fig. 1 b). PCR analysis generated a product of ∼1400 bp, consistent with a monomer length of 2.2 kb (data not show). Sanger sequencing of a clone of this PCR product confirmed the expected arrangement of a 2.2 kb monomer (Fig. 1 b).

### Sequence similarity in *Nic*CL3

**Figure 5:**
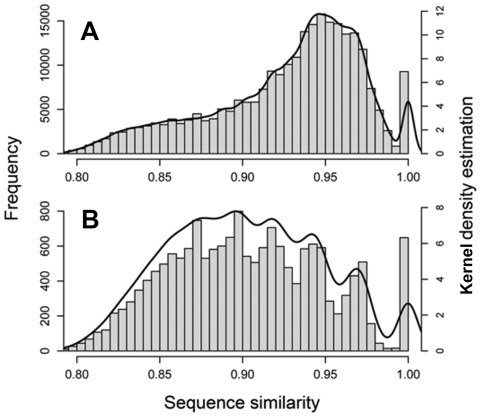
Sequence similarity of *Nic*CL3. Histogram of sequence similarity of *Nic*CL3 derived reads in *N. tomentosiformis* (a) and *N. tabacum* (b). Kernel density estimations are also shown (black line). Note that both species have evidence of sequence amplification and/or homogenisation (peak at sequence similarity of one). There are 6 peaks in *N. tabacum*, perhaps indicative of several independent rounds of ancient amplification and or homogenisation. There are a relatively high proportion of low similarity sequences in *N. tabacum* compared to *N. tomentosiformis.*

In order to detect evidence for rounds of amplification and/or homogenisation of *Nic*CL3, we compared sequence similarity of *Nic*CL3 derived 454 reads in *N. tomentosiformis*, *N. tabacum* and *N. sylvestris*. Reads deriving from *N. tomentosiformis* and *N. tabacum* were analysed separately. In addition, we analysed a dataset consisting of reads from *N. sylvestris* and *N. tomentosiformis* (representing parental additivity). However because there were so few reads from *N. sylvestris* the output was nearly identical to that from *N. tomentosiformis* alone (data not shown). Pair-wise similarity scores for *Nic*CL3 sequences from *N. tabacum* and *N. tomentosiformis* were plotted as frequency distributions and kernel density estimates (Fig. 5). This analysis revealed a peak of identical sequences in both *N. tomentosiformis* and separately in *N. tabacum*. In addition a major peak of reads with sequence similarity close to 0.95 is evident in *N. tomentosiformis*. In *N. tabacum* six separate peaks are visible and the *N. tabacum* genome contains proportionally more reads with lower sequence similarity compared with *N. tomentosiformis* (Fig. S1). A two-sample Wilcoxon test revealed a significant difference (*p*<0.00001) between mean sequence similarity of *Nic*CL3 derived-sequences in *N. tomentosiformis* (0.93) and *N. tabacum* (0.90). We also examined the proportion of sequence reads from *N. tabacum* or *N. tomentosiformis* matching the consensus (*Nic*CL3, contig 8) for each nucleotide along its length (Figure S1 A). We plotted the average proportion of bases identical to the consensus over consecutive 20 bp windows (Figure S1 B). The data indicate that a similar proportion of bases match along the length of the consensus in both species, with the exception of a region towards the end of *Nic*CL3, where the reads are more divergent.

## Discussion

### *Nic*CL3, an abundant repetitive sequence

Data presented here indicate that next generation sequencing, even with low genome coverage, is an effective way to characterise novel repeats and to compare their evolutionary dynamics between related species. We show that one of the most abundant repeats in the *N. tomentosiformis* genome, *Nic*CL3 (Table 1), is predominantly arranged in tandem (Fig. 1 c, Fig. 4), has a unit length of ∼2.2 kb (Fig. 4, Table 2) and is localized in *N. tomentosiformis*, *N. kawakamii*, several synthetic *N. tabacum* lines and natural *N. tabacum* (Fig. 3). However the sequence is not a typical tandem repeat like the *Nicotiana* satellites belonging to the HRS60 family [44] for the following reasons. (1) Typically tandem repeat monomers in angiosperms are ∼180 bp in length [45]. Even the long monomer pSc250 in *Secale cereale* is only 550 bp [46]. (2) Satellite blocks usually occur in long arrays of similar units. However *Nic*CL3 also includes a substantial component that is dispersed (c. 3% in *N. tomentosiformis* and 9% in *N. tabacum* and Fig. 1 c), some of which is associated with *Nic*CL7/30. In *N. tomentosiformis*, Th37 and *N. tabacum Nic*CL3 digestion is almost entirely inhibited when using a methylation sensitive restriction enzyme (Fig. 4). These findings indicate that *Nic*CL3 loci are likely to be heavily methylated. However we observed reads derived from *Nic*CL3 in GSS sequences (obtained after methylation filtration of genomic DNA), although the number of hits was much lower than expected based on 454 abundance estimates. Since the *Nic*CL3 is highly methylated (Fig. 4) it follows that most units were lost by methylation filtration. Rare hits may originate from euchromatic, potentially transcribed parts of the array.

*Nic*CL3 shares sequence similarity with regions previously found flanking NtoEPRV (endogenous pararetrovirus) insertions [42] (Fig. 1 b). The unusually long tandem sequence (2.2 kb) and a small region with similarity to GYPSODE sequences might indicate that *Nic*CL3 includes part of a Ty3/*gypsy* retroelement, that now occurs predominantly in tandem array. Similar compound satellites with long monomers that include sections of retroelement sequences have been described in *Solanum tuberosum* [47] and *Secale cereale* [48].

### Elimination of *Nic*CL3 in synthetic and natural *N. tabacum*

Next generation sequence (using both Illumina and Roche 454) and FISH analysis have revealed the genome of *N. tabacum* to have a much lower abundance of *Nic*CL3 than expected given its abundance in *N. tomentosiformis*, suggesting large-scale losses (Table 1). A reduction in copy number of *Nic*CL3, amounting to thousands of units has also been observed in fourth generation synthetic *N. tabacum* (Th37). Our supposition that *Nic*CL3 has experienced dramatic loss in natural and synthetic lines is evidenced by:

1. One of the *N. tomentosiformis* accession analysed here is the closest known diploid relative of the T-genome of *N. tabacum* and the actual paternal progenitor lineage of Th37 (acc NIC 479/84; reference Murad *et* *al*. [29]), and this accession has *Nic*CL3 in high abundance (Table 1 and Fig. 3).
2. A total of three *N. tomentosiformis* accessions all show similar and strong *Nic*CL3 hybridisation patterns in either FISH (Nee et al. 51771 and NIC 479/84; Fig. 3 and Table 2) and/or Southern blot hybridisation (TW142 and NIC 479/84; Fig. 4 and Table 2).
3. *N. kawakamii* and *N. tomentosiformis* are sister taxa in phylogenetic analysis [26] and both have strong *Nic*CL3 probe binding in FISH and Southern blot analysis (Fig. 3, 4 and Table 2).

Together (1), (2) and (3) indicate *Nic*CL3 was probably abundant in the common ancestor of *N. kawakamii* and *N. tomentosiformis* as well as the true paternal ancestor of *N. tabacum*. Therefore the discrepancy between the expected GP and observed GP in *N. tabacum*, as well as the loss of *Nic*CL3 loci in Th37, is likely to be due to sequence reduction in the allopolyploids rather than expansion in the progenitor post allopolyploidy.

We have shown that, in synthetic *N. tabacum* lines Th37-3, -7 and -14 the number of large blocks of *Nic*CL3 signal is reduced from eight signals to two (Fig. 3 b, d). Several lines of Th37 (3,5,6,7 and 8) show low, but detectable levels of *Nic*CL3 following Southern blot analysis. It is clear that whole loci carrying many thousands of *Nic*CL3 units have been lost from synthetic lines. In addition two synthetic *N. tabacum* lines (Th37-1, 9, Table 2) lack any detectable *Nic*CL3 signal both in Southern blot and FISH analysis indicating that this sequence has been completely (or near completely) eliminated very rapidly indeed – within the first four generations of selfing. We estimate this amounts to the removal of nearly 1% of the Th37 genome in only four generations.

Directional loss of parental sequences has been observed in several synthetic Th37 lines [16], [49], as well as in natural *N. tabacum* [30], [50], where there is a trend for repeats derived from *N. tomentosiformis* to be under-represented [15]. In this paper we have shown that *Nic*CL3 is eliminated or reduced in copy number in synthetic *N. tabacum* lines and is much reduced in copy number in natural *N. tabacum*, suggesting directed mechanisms of removal.

### Mechanisms of *Nic*CL3 loss

The loss of *Nic*CL3 in synthetic *N. tabacum* Th37-1, 3, 8 and 9 cannot be attributed to incomplete chromosomal contribution from *N. tomentosiformis* as GISH to metaphase spreads show the expected number of *N. tomentosiformis*-derived chromosomes (Fig. 3 and Skalicka et al. [16]). Repeats arranged in tandem, for example rDNA, are thought to alter their copy number via unequal crossing-over, although the exact mechanisms are still obscure [51], [52]. Homeologous chromosome pairing has been proposed as a mechanism of sequence and chromosome loss [53], and compelling evidence exists for such chromosomal rearrangements in synthetic *Brassica* hybrids [54], [55] and recently formed *Tragopogon* allopolyploids [56], [57]. However Salina *et* *al.*, [58] suggested that changes in copy number of tandem repeats Spelt1 and Spelt52 in synthetic wheats, were not a consequence of intergenomic recombination during meiosis, as lines with or without the *Ph1* locus show similar patterns of copy number change (*Ph1* mutants have increased frequency of homeologous pairing). Similarly in *Nicotiana* there is no evidence for extensive homeologous pairing [9], [27], [59], and so an alternative explanation is needed. Striking sequence homologies exist between different chromosomes of the same species: essentially the same repeats form large blocks of heterochromatin on multiple chromosomes of both S and T genomes (this study and Lim *et* *al*. [9]). Hence, it is possible that recombination between large blocks at homologous and non-homologous loci carrying *Nic*CL3 may explain its elimination. Indeed the higher proportion of solo HSPs in paired-end data in *N. tabacum* compared to *N. tomentosiformis* is consistent with the preferential loss of the tandem repeated component of *Nic*CL3 in the allopolyploid (Fig. 1 c).

The outcome of such process would be the generation of chromosomes with either extremely large arrays and/or chromosomes with large deletions of repeats. Indeed if small deletions within the unit were responsible for lowering the genome proportion of *Nic*CL3 in *N. tabacum*, then one might expect to see a smear towards smaller molecular weight fragments in Southern blot analysis (Fig. 4), however this was not observed. Instead the relatively sharp bands suggest that the removal of whole units within the tandem array is responsible for the reduced abundance of *Nic*CL3 in *N. tabacum*.

Recombination mechanisms are thought to be responsible for homogenisation of sequences arranged in tandem and there is evidence that this has occurred in *Nic*CL3 (Fig. 5). Both *N. tabacum* and *N. tomentosiformis* have a peak in the number of sequences with a nucleotide similarity of one. *Nicotiana tabacum* has a series of peaks each with progressively less sequence similarity (Fig. 5), perhaps indicative of more ancient rounds of homogenisation. It is possible that these events coincide with *Nic*CL3 unit loss.

We examined the possibility that different regions of *Nic*CL3 may be more variable than others, a pattern that would explain the series of peaks in Fig. 5. However analysis of the sequence similarity of reads against the consensus failed to provide any evidence of such a pattern in either *N tabacum* or *N. tomentosiformis* (Fig. S1). Hence a hypothesis of repeated rounds of sequence homogenisation seems a better explanation for the series of peaks.

Our study is significant in providing evidence of multiple large-scale deletions, occurring repeatedly in both natural and synthetic material. This has resulted in the removal of almost all of the continuous arrays of *Nic*CL3 in *N. tabacum*. We have hypothesized that the loss is most likely the result of multiple unequal recombination events between tandem components of *Nic*CL3 and the maintenance of dispersed units of *Nic*CL3 suggests they are more stable than those in tandem array.

## Supporting Information

Figure S1 **Sequence similarity of BLASTn hits to the consensus of** ***Nic*** **CL3 (contig 8) calculated by examining the proportion of hits that match the consensus over a given nucleotide.** (a) All the data points for each nucleotide in the consensus and (b) the data averaged over consecutive 20 bp windows.
(TIF)

Table S1 **Dataset size and average read length for the four Illumina runs used in this analysis following the removal of plastid sequences.**
(DOCX)

## References

[1] Adams KL, Wendel JF (2005). Polyploidy and genome evolution in plants.. Current Opinion in Plant Biology.

[2] Leitch AR, Leitch IJ (2008). Perspective – Genomic plasticity and the diversity of polyploid plants.. Science.

[3] Soltis DE, Albert VA, Leebens-Mack J, Bell CD, Paterson AH (2009). Polyploidy and angiosperm diversification.. American Journal of Botany.

[4] Wendel JF (2000). Genome evolution in polyploids.. Plant Molecular Biology.

[5] Jiao Y, Wickett NJ, Ayyampalayam S, Chanderbali AS, Landherr L (2011). Ancestral polyploidy in seed plants and angiosperms.. Nature.

[6] Chen ZJ, Ni ZF (2006). Mechanisms of genomic rearrangements and gene expression changes in plant polyploids.. Bioessays.

[7] Comai L, Madlung A, Josefsson C, Tyagi A (2003). Do the different parental ‘heteromes’ cause genomic shock in newly formed allopolyploids?. Philosophical Transactions of the Royal Society of London Series B-Biological Sciences.

[8] Feldman M, Levy AA (2009). Genome evolution in allopolyploid wheat-a revolutionary reprogramming followed by gradual changes.. Journal of Genetics and Genomics.

[9] Lim KY, Matyasek R, Kovarik A, Leitch AR (2004). Genome evolution in allotetraploid *Nicotiana*.. Biological Journal of the Linnean Society.

[10] Liu B, Wendel JF (2003). Epigenetic phenomena and the evolution of plant allopolyploids.. Molecular Phylogenetics and Evolution.

[11] Matyasek R, Tate JA, Lim YK, Srubarova H, Koh J (2007). Concerted evolution of rDNA in recently formed *Tragopogon* allotetraploids is typically associated with an inverse correlation between gene copy number and expression.. Genetics.

[12] Shaked H, Kashkush K, Ozkan H, Feldman M, Levy AA (2001). Sequence elimination and cytosine methylation are rapid and reproducible responses of the genome to wide hybridization and allopolyploidy in wheat.. Plant Cell.

[13] Khasdan V, Yaakov B, Kraitshtein Z, Kashkush K (2010). Developmental timing of DNA elimination following allopolyploidization in wheat.. Genetics.

[14] Leitch IJ, Hanson L, Lim KY, Kovarik A, Chase MW (2008). The ups and downs of genome size evolution in polyploid species of *Nicotiana* (Solanaceae).. Annals of Botany.

[15] Renny-Byfield S, Chester M, Kovařík A, Le Comber SC, Grandbastien M-A (2011). Next generation sequencing reveals genome downsizing in allotetraploid *Nicotiana tabacum*, predominantly through the elimination of paternally derived repetitive DNAs.. Molecular Biology and Evolution.

[16] Skalicka K, Lim KY, Matyasek R, Matzke M, Leitch AR (2005). Preferential elimination of repeated DNA sequences from the paternal, *Nicotiana tomentosiformis* genome donor of a synthetic, allotetraploid tobacco.. New Phytologist.

[17] Margulies M, Egholm M, Altman WE, Attiya S, Bader JS (2005). Genome sequencing in microfabricated high-density picolitre reactors.. Nature.

[18] Hribova E, Neumann P, Matsumoto T, Roux N, Macas J (2010). Repetitive part of the banana (*Musa acuminata*) genome investigated by low-depth 454 sequencing.. BMC Plant Biology.

[19] Macas J, Neumann P, Navratilova A (2007). Repetitive DNA in the pea (*Pisum sativum* L.) genome: comprehensive characterization using 454 sequencing and comparison to soybean and *Medicago truncatula*.. BMC Genomics.

[20] Swaminathan K, Varala K, Hudson ME (2007). Global repeat discovery and estimation of genomic copy number in a large, complex genome using a high-throughput 454 sequence survey.. BMC Genomics.

[21] Wicker T, Taudien S, Houben A, Keller B, Graner A (2009). A whole-genome snapshot of 454 sequences exposes the composition of the barley genome and provides evidence for parallel evolution of genome size in wheat and barley.. The Plant Journal.

[22] Macas J, Kejnovsky E, Neumann P, Novak P, Koblizkova A (2011). Next generation sequencing-based analysis of repetitive DNA in the model dioecious plant *Silene latifolia*.. PLoS One.

[23] Burk LG (1973). Partial self-fertility in theoretical amphiploid progenitor of *N. tabacum* Journal of Heredity.

[24] Skalicka K, Lim KY, Matyasek R, Koukalova B, Leitch AR (2003). Rapid evolution of parental rDNA in a synthetic tobacco allotetraploid line.. American Journal of Botany.

[25] Lim KY, Souckova-Skalicka K, Sarasan V, Clarkson JJ, Chase MW (2006). A genetic appraisal of a new synthetic *Nicotiana tabacum* (Solanaceae) and the Kostoff synthetic tobacco.. American Journal of Botany.

[26] Kelly LJ, Leitch AR, Clarkson JJ, Hunter RB, Knapp S (2010). Intragenic recombination events and evidence for hybrid speciation in *Nicotiana* (*Solanaceae*).. Molecular Biology and Evolution.

[27] Goodspeed T (1954). The genus *Nicotiana*.. Chron Bot.

[28] Knapp S, Chase MW, Clarkson JJ (2004). Nomenclatural changes and a new sectional classification in *Nicotiana* (Solanaceae).. Taxon.

[29] Murad L, Lim KY, Christopodulou V, Matyasek R, Lichtenstein CP (2002). The origin of tobacco's T genome is traced to a particular lineage within *Nicotiana tomentosiformis* (Solanaceae).. American Journal of Botany.

[30] Petit M, Lim KY, Julio E, Poncet C, de Borne FD (2007). Differential impact of retrotransposon populations on the genome of allotetraploid tobacco (*Nicotiana tabacum*).. Molecular Genetics and Genomics.

[31] Novak P, Neumann P, Macas J (2010). Graph-based clustering and characterization of repetitive sequences in next-generation sequencing data.. BMC Bioinformatics.

[32] Jurka J, Kapitonov VV, Pavlicek A, Klonowski P, Kohany O (2005). Repbase update, a database of eukaryotic repetitive elements.. Cytogenetic and Genome Research.

[33] Altschul SF, Gish W, Miller W, Myers EW, Lipman DJ (1990). Basic local alignment search tool.. Journal of Molecular Biology.

[34] R Development Core Team (2010). R: A language and environment for statistical computing..

[35] Chester M, Sykorova E, Fajkus J, Leitch AR (2010). Single Integration and Spread of a Copia-Like Sequence Nested in rDNA Intergenic Spacers of *Allium cernuum* (Alliaceae).. Cytogenetic and Genome Research.

[36] Sun Y, Skinner DZ, Liang GH, Hulbert SH (1994). Phylogenetic analysis of *Sorghum* and related taxa using internal transcribed spacers of nuclear ribosomal DNA Theoretical and Applied Genetics.

[37] Lim KY, Leitch IJ, Leitch AR (1998). Genomic characterisation and the detection of raspberry chromatin in polyploid *Rubus*.. Theoretical and Applied Genetics.

[38] Lim KY, Kovarik A, Matyasek R, Chase MW, Knapp S (2006). Comparative genomics and repetitive sequence divergence in the species of diploid *Nicotiana* section *Alatae*.. Plant Journal.

[39] Kovarik A, Koukalova B, Lim KY, Matyasek R, Lichtenstein CP (2000). Comparative analysis of DNA methylation in tobacco heterochromatic sequences.. Chromosome Research.

[40] Sambrook J, Russell DW (2001). Molecular Cloning: A Laboratory Manual..

[41] Bennett MD, Leitch IJ (2005). Angiosperm DNA C-values database.

[42] Matzke M, Gregor W, Mette MF, Aufsatz W, Kanno T (2004). Endogenous pararetroviruses of allotetraploid *Nicotiana tabacum* and its diploid progenitors, *N. sylvestris* and *N. tomentosiformis*.. Biological Journal of the Linnean Society.

[43] Lim KY, Matyasek R, Lichtenstein CP, Leitch AR (2000). Molecular cytogenetic analyses and phylogenetic studies in the *Nicotiana* section *Tomentosae*.. Chromosoma.

[44] Koukalova B, Moraes AP, Renny-Byfield S, Matyasek R, Leitch AR (2010). Fall and rise of satellite repeats in allopolyploids of *Nicotiana* over c. 5 million years.. New Phytologist.

[45] Heslop-Harrison JS, Schwarzacher T (2011). Organisation of the plant genome in chromosomes.. Plant Journal.

[46] Vershinin AV, Schwarzacher T, Heslop-Harrison JS (1995). The large-scale genomic organization of repetitive DNA families at the telomeres of rye chromsomes.. Plant Cell.

[47] Tek AL, Song JQ, Macas J, Jiang JM (2005). Sobo, a recently amplified satellite repeat of potato, and its implications for the origin of tandemly repeated sequences.. Genetics.

[48] Langdon T, Seago C, Jones RN, Ougham H, Thomas H (2000). De novo evolution of satellite DNA on the rye B chromosome.. Genetics.

[49] Petit M, Guidat C, Daniel J, Denis E, Montoriol E (2010). Mobilization of retrotransposons in synthetic allotetraploid tobacco.. New Phytologist.

[50] Volkov RA, Borisjuk NV, Panchuk, II, Schweizer D, Hemleben V (1999). Elimination and rearrangement of parental rDNA in the allotetraploid *Nicotiana tabacum*.. Molecular Biology and Evolution.

[51] Eickbush TH, Eickbush DG (2007). Finely orchestrated movements: Evolution of the ribosomal RNA genes.. Genetics.

[52] Ganley ARD, Kobayashi T (2007). Highly efficient concerted evolution in the ribosomal DNA repeats: Total rDNA repeat variation revealed by whole-genome shotgun sequence data.. Genome Research.

[53] Jones RN, Hegarty M (2009). Order out of chaos in the hybrid plant nucleus.. Cytogenetic and Genome Research.

[54] Gaeta RT, Pires JC, Iniguez-Luy F, Leon E, Osborn TC (2007). Genomic changes in resynthesized *Brassica napus* and their effect on gene expression and phenotype.. Plant Cell.

[55] Szadkowski E, Eber F, Huteau V, Lode M, Huneau C (2010). The first meiosis of resynthesized *Brassica napus*, a genome blender.. New Phytologist.

[56] Kovarik A, Pires JC, Leitch AR, Lim KY, Sherwood AM (2005). Rapid concerted evolution of nuclear ribosomal DNA in two *Tragopogon* allopolyploids of recent and recurrent origin.. Genetics.

[57] Lim KY, Soltis DE, Soltis PS, Tate J, Matyasek R (2008). Rapid chromosome evolution in recently formed polyploids in *Tragopogon* (Asteraceae).. PLoS ONE.

[58] Salina EA, Numerova OM, Ozkan H, Feldman M (2004). Alterations in subtelomeric tandem repeats during early stages of allopolyploidy in wheat.. Genome.

[59] Lim KY, Kovarik A, Matyasek R, Bezdek M, Lichtenstein CP (2000). Gene conversion of ribosomal DNA in *Nicotiana tabacum* is associated with undermethylated, decondensed and probably active gene units.. Chromosoma.

