# Supplementary figures and images for: Independent, Rapid and Targeted Loss of Highly Repetitive DNA in Natural and Synthetic Allopolyploids of Nicotiana tabacum
Source: PLoS One. 2012 May 14;7(5):e36963. doi: 10.1371/journal.pone.0036963 (PMC3351487; doi:10.1371/journal.pone.0036963)

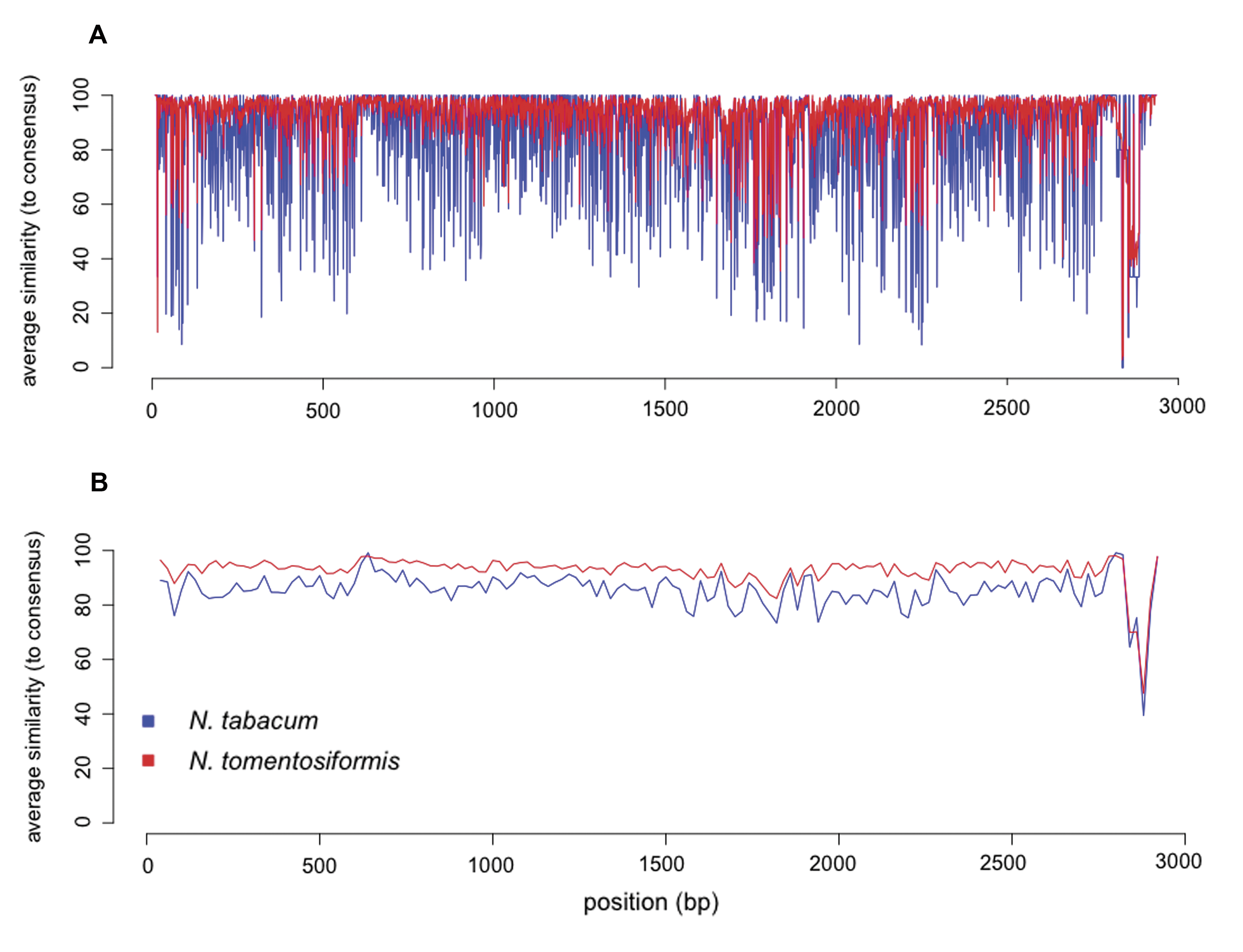

Supplement: Figure S1 — Sequence similarity of BLASTn hits to the consensus of Nic CL3 (contig 8) calculated by examining the proportion of hits that match the consensus over a given nucleotide. (a) All the data points for each nucleotide in the consensus and (b) the data averaged over consecutive 20 bp windows. (TIF) [file pone.0036963.s001.tif]
