# Supplementary material for: Independent, Rapid and Targeted Loss of Highly Repetitive DNA in Natural and Synthetic Allopolyploids of Nicotiana tabacum
Source: PLoS One. 2012 May 14;7(5):e36963. doi: 10.1371/journal.pone.0036963 (PMC3351487; doi:10.1371/journal.pone.0036963)
Supplement: Table S1 — Dataset size and average read length for the four Illumina runs used in this analysis following the removal of plastid sequences. (DOCX) [file pone.0036963.s002.docx]

| Species | Average read length (bp) | Dataset Size (bp) |
| --- | --- | --- |
| *N. tomentosiformis* | 96.7 | 1,390,138,328 |
| *N. sylvestris* | 96.7 | 1,259,648,345 |
| *N. tabacum* (SR1) | 96.7 | 2,046,389,071 |
| TR1-A (synthetic tobacco) | 96.5 | 2,318,504,049 |

**Supplementary Table 1**: Dataset size and average read length for the four Illumina runs used in this analysis following the removal of plastid sequences
